# Genome-wide distribution of genetic diversity and linkage disequilibrium in elite sugar beet germplasm

**DOI:** 10.1186/1471-2164-12-484

**Published:** 2011-10-04

**Authors:** Jinquan Li, Ann-Katrin Lühmann, Knuth Weißleder, Benjamin Stich

**Affiliations:** 1 Max Planck Institute for Plant Breeding Research, Carl-von-Linné-Weg 10, 50829 Köln, Germany; 2 KWS SAAT AG, Grimsehlstr. 31, 37555 Einbeck, Germany

## Abstract

**Background:**

Characterization of population structure and genetic diversity of germplasm is essential for the efficient organization and utilization of breeding material. The objectives of this study were to (i) explore the patterns of population structure in the pollen parent heterotic pool using different methods, (ii) investigate the genome-wide distribution of genetic diversity, and (iii) assess the extent and genome-wide distribution of linkage disequilibrium (LD) in elite sugar beet germplasm.

**Results:**

A total of 264 and 238 inbred lines from the yield type and sugar type inbreds of the pollen parent heterotic gene pools, respectively, which had been genotyped with 328 SNP markers, were used in this study. Two distinct subgroups were detected based on different statistical methods within the elite sugar beet germplasm set, which was in accordance with its breeding history. MCLUST based on principal components, principal coordinates, or lapvectors had high correspondence with the germplasm type information as well as the assignment by STRUCTURE, which indicated that these methods might be alternatives to STRUCTURE for population structure analysis. Gene diversity and modified Roger's distance between the examined germplasm types varied considerably across the genome, which might be due to artificial selection. This observation indicates that population genetic approaches could be used to identify candidate genes for the traits under selection. Due to the fact that *r*^2 ^*>*0.8 is required to detect marker-phenotype association explaining less than 1% of the phenotypic variance, our observation of a low proportion of SNP loci pairs showing such levels of LD suggests that the number of markers has to be dramatically increased for powerful genome-wide association mapping.

**Conclusions:**

We provided a genome-wide distribution map of genetic diversity and linkage disequilibrium for the elite sugar beet germplasm, which is useful for the application of genome-wide association mapping in sugar beet as well as the efficient organization of germplasm.

## Background

Sugar beet (*Beta vulgaris *subsp. *vulgaris*) is a member of the family *Amaranthaceae *[1]. It is an important crop for sucrose production in the temperate climate zone, which accounts for about one quarter to one third of the worldwide sugar production [2]. Sugar beet is a diploid species with n = nine chromosomes and a haploid genome size of 758 Mb [3]. Physical mapping and sequencing of the sugar beet genome is in progress [4].

At present, hybrid varieties account for most of the sugar beet production. Seed and pollen parent heterotic pools are the basic material for hybrid breeding [5], where the former consists of monogerm germplasm and the latter of multigerm germplasm (e.g. [6]). Due to the strong negative correlation between root yield and sugar content in sugar beet [7], the germplasm of the individual heterotic pools is usually classified as yield type (with emphasis on root yield), sugar type (with emphasis on sugar content), or normal type (intermediate in both characters)[8]. The relatively independent development of these different types of germplasm through decades might have resulted in divergent populations. Such information, however, is not available for sugar beet.

Molecular markers reflect the actual level of genetic variation existing among genotypes at the DNA level and therefore have been widely applied in population genetics research. In beets, the most frequently used class of molecular markers are microsatellites or simple sequence repeat (SSR) markers as they are highly polymorphic and co-dominantly inherited (e.g. [9]). The recent advances in genomic technologies, however, have provided with single nucleotide polymorphism (SNP) markers a powerful tool for a more direct analysis of sequence-based polymorphisms [10]. They are the most abundant class of sequence variability in the genome, co-dominantly inherited, easily automated and, thus, appropriate for high throughput analyses [11]. Therefore, they are now the marker system of choice for various crop species such as maize [12], rice [13], barley [14], and soybean [15]. For sugar beet, a few studies have been carried out on the identification of SNPs [16,1]. No earlier study, however, evaluated SNP markers with respect to their usefulness to characterize genetic diversity and population structure in elite sugar beet germplasm. Furthermore, no information is available on the number of SNPs required for such analyses.

Various methods have been proposed for examining population structure. One of the most frequently used methods is STRUCTURE, a model-based approach to assign individuals to subgroups [17]. Furthermore, principal component analysis (PCA) and principal coordinate analysis (PCoA) are considered favourable for uncovering population structure [18,19]. Laplacian eigenfunctions (LAP), as a weighted PCA, were recently reported to describe population structure [20]. Another model-based approach, MCLUST, was reported being appropriate for determining the clusters and membership simultaneously without genetic assumptions [21]. Despite that advantages and disadvantages of the different methods are known, few empirical comparisons are available in a plant genetics context.

The identification of genes underlying phenotypic variation can be performed in two different directions: (i) from phenotype to genotype, which is used in quantitative genetics approaches and (ii) from genotype to phenotype, which evaluates signatures of selection [22]. High density SNP markers allow to evaluate the genomic changes that occurred by artificial selection during breeding and have the potential to help identifying likely targets of past selection. To our knowledge, however, such analyses have not been performed for sugar beet yet.

The potential of using association mapping approaches in sugar beet has come to the forefront (e.g. [23,24]). This approach depends on the extent and distribution of linkage disequilibrium (LD). Several studies examining LD in beets are available, where these were based on a relatively few RFLP, SSR, RAPD or AFLP makers ([25-27,9,6]). However, to the best of our knowledge, no earlier study examined the extent and genome-wide distribution of LD in elite sugar beet germplasm with a high number of genome-wide distributed markers.

The objectives of this study were to (i) explore the patterns of population structure in the pollen parent heterotic pool using different methods, (ii) investigate the genome-wide distribution of genetic diversity, and (iii) assess the extent and genome-wide distribution of LD in elite sugar beet germplasm.

## Methods

### Plant materials and molecular markers

A total of 502 diploid sugar beet inbreds from the pollen parent heterotic pool were examined in this study. Among them, 264 accessions were yield types and 238 sugar types. All plant materials used in this study are proprietary to KWS SAAT AG (Einbeck, Germany). All 502 sugar beet inbreds were genotyped by KWS SAAT AG, following standard protocols, with 328 SNPs markers, which were distributed across the genome. A total of 26, 33, 41, 35, 40, 42, 39, 32, and 40 of these markers map to linkage group A to I, respectively (unpublished data). This data set comprises no inbreds or markers with more than 20% missing data.

### Statistical analyses

The model-based approach implemented in software package STRUCTURE [17] was used to examine population structure. STRUCTURE was run for *K *= 1-10 subgroups using the linkage model neglecting prior information. Each run consisted of a burn-in period of 100,000 steps followed by 100,000 Monte Carlo Markov Chain replicates, assuming that allele frequencies are uncorrelated across clusters. Five replications were performed for each *K *value. To determine the most probable value of *K*, an ad hoc criterion was used [28]. That run of the estimated number of subgroups showing the maximum likelihood was used to assign inbreds with membership probabilities surpassing a certain threshold (i.e. maximum probabilities among the subgroups, membership probabilities of 0.60, 0.70, and 0.80) to subgroups. The results from STRUCTURE were displayed by DISTRUCT software [29].

The allele frequencies at each marker and for each inbred were calculated and used for PCA analyses [18]. The number of significant PCA eigenvalues was tested by Eigenanalysis (cf. [30]). Furthermore, the modified Rogers distance (MRD) was calculated [31]. PCoA [19] based on MRD estimates between pairs of inbred lines was performed. In addition, we used LAP [20] to reveal the population structure, where the threshold of correlation coefficients *eps *was set to 0.8. Finally, the model-based approach MCLUST was used to determine the number of subgroups as well as to provide the membership probabilities [21]. Due to the large number of dimensions (328 markers), MCLUST analysis was performed on 1-150 PCA components, PCoA coordinates, or LAP lapvectors, respectively. Models for 1 to 15 subgroups were examined. The correspondence between the inbreds' assignment by MCLUST and STRUCTURE and the germplasm type information were compared.

In order to determine the number of SNPs required to detect the underlying population structure, a resampling analysis was performed. In each of 100 repetitions, subsets of the markers (9 to 252 by 9 grad) were either randomly selected (random sampling) or sampled in such a way that the selected markers were equally distributed across the genome (stratified sampling) [12]. Based on the selected markers, PCA was performed for all the inbreds and 10 PCA components were used for MCLUST analysis. The correspondence between the inbreds' assignment by MCLUST based on the entire set of 328 SNPs and different resampling subsets was compared. The MRD was calculated for each pair of inbreds based on the selected SNP markers and the coefficient of variation (*CV*) across all 100 repetitions was calculated. Furthermore, subsets of the markers (9 to 252 by 9 grad) showing the highest polymorphic information content (PIC) or MRD between the two germplasm types were selected. Based on the selected markers, PCA was performed as described above. The correspondence between the inbreds' assignment by MCLUST based on the entire set of 328 SNPs and the SNP subsets was compared.

Gene diversity was calculated for the yield type as well as sugar type inbreds for each marker separately. Similarly, MRD between yield type and sugar type inbreds was calculated on an individual marker basis.

The squared correlation of allele frequencies (*r*^2^) at two SNP loci was calculated to measure the LD level. This measure was chosen as it can be interpreted as the proportion of variance which the allele frequency of the first marker explains of the allele frequency of the second marker [32]. The 95% quantile of *r*^2 ^for unlinked loci pairs was used as significance threshold for the linked loci pairs. A nonlinear regression of *r*^2 ^vs. the genetic map distance (cM) was performed according to [33]. The expectation of *r*^2 ^between adjacent sites is: $E\left(r^{2}\right)=\left[\frac{10+C}{\left(2+C\right)\left(11+C\right)}\right]\left[1+\frac{\left(3+C\right)\left(12+12C+C^{2}\right)}{n\left(2+C\right)\left(11+C\right)}\right]$[34], where *C *= 4*Ner*, r the recombination rate, *n *the sample size, and *Ne *the effective population size. The average $r^{2}\left(\bar{r^{2}}\right)$ at binned genetic distances was calculated. Furthermore, the $\bar{r^{2}}$ for all linked loci pairs within 5 cM segments across the genome was calculated. All LD analyses were performed for the entire germplasm set, yield type, and sugar type inbreds.

If not stated differently, all analyses were performed with the statistical software R [35].

## Results

**Figure 1:**
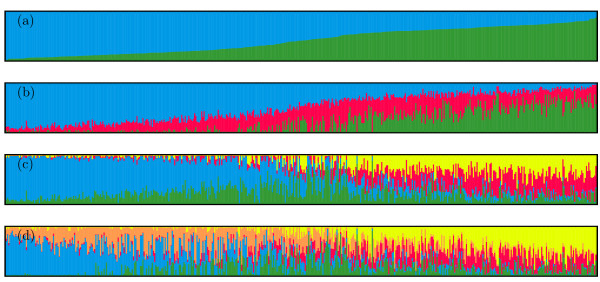
**Membership probability of assigning inbreds of the entire germplasm set to (a) two, (b) three, (c) four, and (d) five subgroups**. The height of each bar represents the probability of each inbred belonging to different subgroups. The inbreds were sorted according to their membership probability in (a).

The log likelihood revealed by STRUCTURE increased gradually from *K *= 1 to *K *= 10 and showed no obvious optimum (Additional file 1). In contrast, the maximum of the ad hoc measure Δ*K *was observed for *K *= 2. Based on the membership probability thresholds of 0.80, 0.70, and 0.60, 36%, 60%, and 84% of the inbreds of the entire germplasm set could be assigned to two subgroups, respectively. With the maximum membership probability criterion, the assignment by STRUCTURE showed for 94.4% of the inbreds correspondence with the germplasm type information (Figure 1, Additional file 2).

PCA, PCoA, as well as LAP revealed two distinct clusters for the entire germplasm set (Additional file 2). The first and second principal component explained 22.7% and 5.4% of the molecular variance, respectively. In PCoA based on MRD estimates between all pairs of sugar beet inbreds, the first two principal coordinates explained 23.2% and 5.5% of the molecular variance. In addition, the first and second lapvectors of LAP explained 14.6% and 3.5% of the molecular variance, respectively.

**Figure 2:**
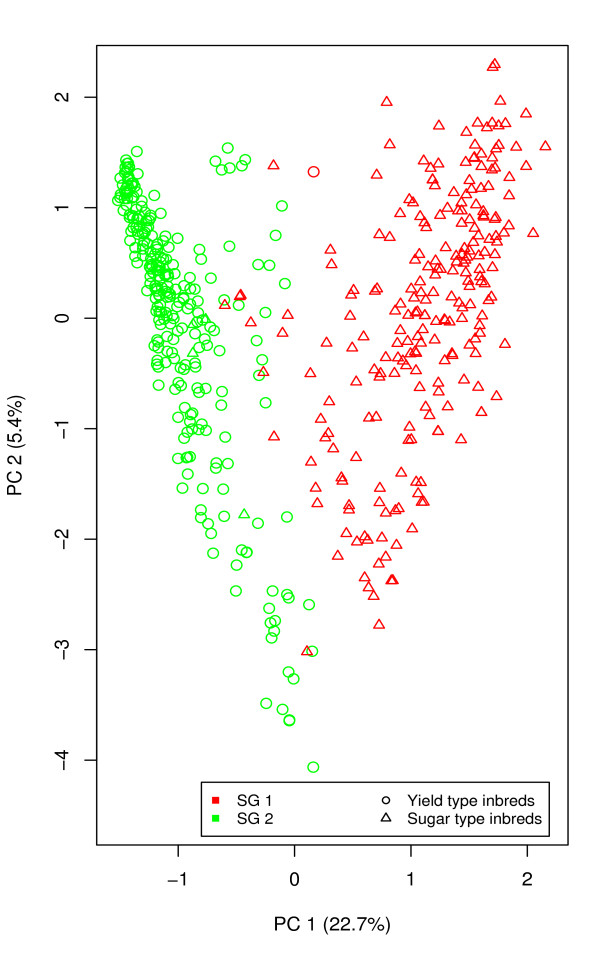
**Principal component analysis of the 502 sugar beet inbreds**. PC 1 and PC 2 refer to the first and second principal component. The numbers in parentheses refer to the proportion of variance explained by the principal components. Colors identify different subgroups (SG) assigned by MCLUST based on 10 principal components and symbols identify different germplasm types.

The number of subgroups identified by MCLUST based on 1-150 PCA components varied from 1 to 9, while the number for 1-150 PCoA coordinates or LAP lapvectors varied from 2 to 9 (Additional file 3). When the number of subgroups was set to two, MCLUST analysis based on 8-50 PCA components, 8-50 PCoA coordinates, and 1-100 LAP lapvectors showed with *>*90% a high correspondence of assignment with the germplasm type information (Figure 2, Additional file 4).

**Figure 3:**
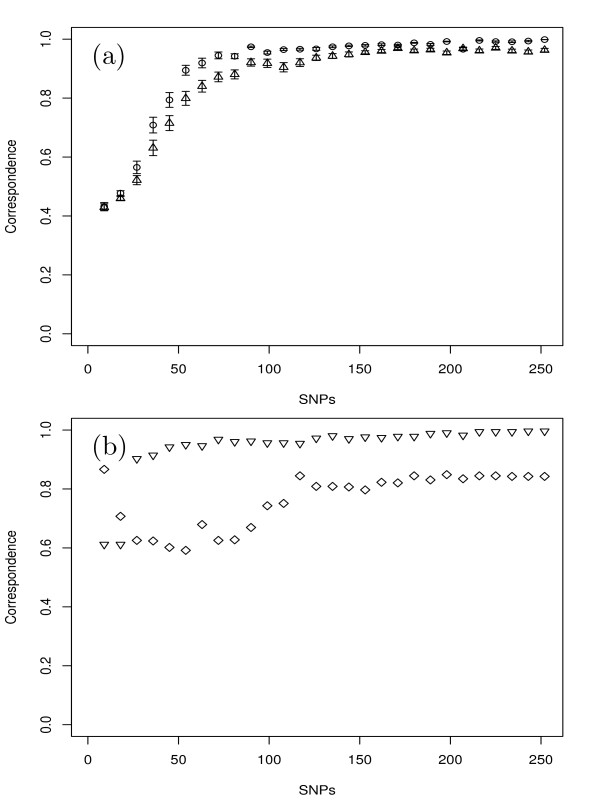
**Correspondence between the assignment of all 502 inbreds based on the entire set of 328 SNPs by applying MCLUST on 10 principal components and different subsets of SNP markers selected (a) at random (triangles point-up) or stratified (circles) with 100 replications, and (b) showing high modified Roger's distance (MRD) between sugar and yield type inbreds (square) or highest polymorphic information content (triangles point down)**. The vertical lines at each point indicate the standard error. For details see Materials and Methods.

**Figure 4:**
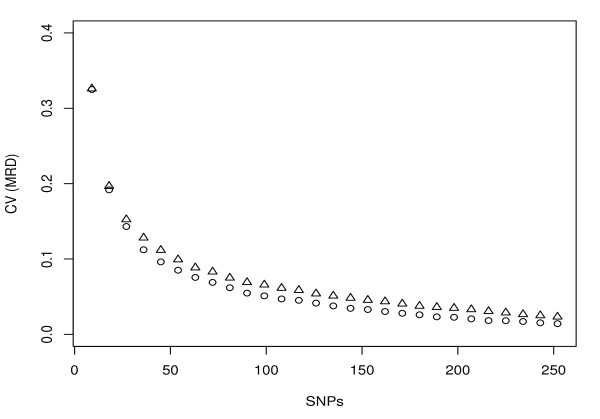
**Coefficient of variation of modified Roger's distance (MRD) estimates among all pairs of inbreds assessed by random (triangles) and stratified (circles) resampling with 100 replications**. For details see Materials and Methods.

MCLUST was used to assign inbreds based on different resampling subsets of all SNPs to clusters, where the correspondence to the clustering using all SNPs improved with increasing number of SNP markers. When the number of SNP markers reached about 100, not much higher correspondence could be obtained by further increasing the number of SNPs (Figure 3). Similarly, the *CV *of MRD among all pairs of inbreds decreased as the number of SNP markers increased (Figure 4). When the number of SNP markers reached about 100, not much lower *CV *of MRD could be obtained by further increasing the number of SNPs. The stratified resampling strategy revealed a slightly higher correspondence and lower *CV *compared to the random resampling strategy. Furthermore, MCLUST analysis based on SNP markers selected for their high PIC values revealed a higher correspondence to the clustering using all SNPs than based on the SNP markers selected for a high MRD between yield and sugar types as well as based on the above mentioned stratified and random resampling strategy (Figure 3).

The average gene diversity of the entire germplasm set, yield type, and sugar type inbreds were 0.338, 0.199, and 0.365, respectively. Gene diversity for yield type and sugar type inbreds varied across the genome (Additional file 5). For most genome regions, the sugar type inbreds showed a higher gene diversity than the yield type inbreds. However, for a few regions, the opposite was true. The average MRD among all inbreds was 0.562, and the MRD between yield type and sugar type inbreds was 0.311. A different degree of divergence between these two germplasm types was observed across the genome (Additional file 6).

**Figure 5:**
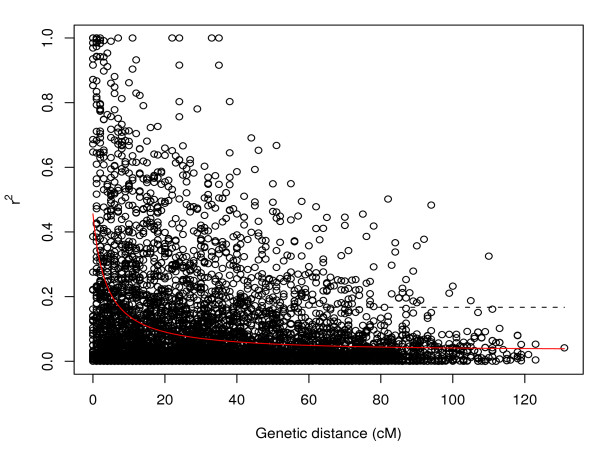
**Plot of linkage disequilibrium measured as squared correlation of allele frequencies (***r*^2^**) against genetic map distance (cM) between linked loci pairs in the entire germplasm set**. The red line is the nonlinear regression trend line of *r*^2 ^vs. genetic map distance. The dashed line indicates the 95% quantile of *r*^2 ^between unlinked loci pairs.

**Figure 6:**
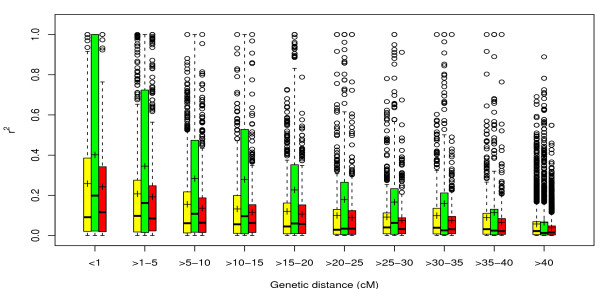
**Boxplot of linkage disequilibrium measured as squared correlation of allele frequencies (***r*^2^**) at binned genetic map distances (cM) for the entire germplasm set (yellow), yield (green), and sugar type inbreds (red)**.

The 95% quantile of *r*^2 ^values for unlinked loci pairs in the entire germplasm set, yield type, and sugar type inbreds was 0.167, 0.117, and 0.071, respectively (Table 1). A total of 18.97%, 31.84%, and 32.02% of linked loci pairs in the entire germplasm set, yield type and sugar type inbreds, respectively, showed an *r*^2 ^level higher than the $r_{Q95}^{2}$ of unlinked loci pairs. A total of 0.93%, 6.22%, and 0.74% of *r*^2 ^values between linked loci pairs in the germplasm sets were larger than 0.8. LD decayed to $r_{Q95}^{2}$ of unlinked loci pairs within 7.4 cM, 45.1 cM, and 20.6 cM for the entire germplasm set, yield type, and sugar type inbreds, respectively (Figure 5, Additional file 7). The $\bar{r^{2}}$ between marker loci within binned genetic distances decreased as the genetic distance intervals increased (Figure 6). When the intervals reached 15-20 cM, the $\bar{r^{2}}$ reached a plateau. For all intervals, the yield type inbreds showed higher *r*^2 ^values than the entire germplasm set and sugar type inbreds, while the latter two showed similar trends. The $\bar{r^{2}}$ for all linked loci pairs within 5 cM segments varied considerably across the genome (Additional file 8). The effective population size for the entire germplasm set, yield type, and sugar type inbreds were 52.7, 21.2, and 72.7, respectively, and these values varied considerably between the different linkage groups (Table 2).

**Table 1:** The $\bar{r^{2}}$, 95% quantile of *r*^2 ^for unlinked loci pairs $\left(r_{Q95}^{2}\right)$, percentage of *r*^2 ^values larger than *r*^2^_*Q*95 _or 0.8 for linked and unlinked loci pairs for the entire germplasm set, the yield type, and sugar type inbreds.

Germplasm Group		Linked			Unlinked		
			
	*N*	$\bar{r^{2}}$	$\%>r_{Q95}^{2}$	% *>*0.8	$\bar{r^{2}}$	*r*^2^*_Q_*_95_	% *>*0.8
Yield type inbreds	264	0.165	31.84	6.22	0.027	0.117	0.03
Sugar type inbreds	238	0.083	32.02	0.74	0.019	0.071	0.00
Entire germplasm set	502	0.101	18.97	0.93	0.040	0.167	0.00

*N *is the sample size.

**Table 2:** The effective population size (*Ne*) of the entire germplasm set, yield type, and sugar type inbreds for each linkage group (A-I).

Germplasm group	A	B	C	D	E	F	G	H	I	All
Yield type inbreds	47.1	30.7	16.8	31.6	15.5	12.3	16.5	29.2	23.6	21.2
Sugar type inbreds	210.7	84.4	91.8	83.3	36.0	92.7	48.2	66.2	81.6	72.7
Entire germplasm set	137.4	68.0	62.9	89.2	23.0	52.8	28.3	57.3	80.0	52.7

## Discussion

### Comparison of different approaches for detecting population structure

Knowledge about the patterns of population structure is essential for efficient germplasm organization. Therefore, various approaches have been developed for this purpose. The method implemented in the software STRUCTURE is one of the most frequently used approaches. However, when dealing with thousands of individuals and markers, the high computational requirements of STRUCTURE analyses make it impractical [36]. Instead, PCA, PCoA, as well as LAP have the potential to extract the fundamental structure of a dataset without assuming any population genetic model [18,19]. Furthermore, as these methods are not computationally intensive, they might be possible alternatives for detecting population structure.

These approaches, however, do not allow to make directly statistical inferences about the number of subgroups. Furthermore, the assignment of inbreds to subgroups is not defined. MCLUST, however, could determine the numbers of subgroup as well as the cluster membership probability simultaneously without genetic assumptions [21]. Nevertheless, MCLUST applied directly to the raw marker data had in our study only a low power to identify population structure (data not shown). This might be due to the fact that many markers explain a small part of the population structure information. To overcome this problem, MCLUST was applied in our study on principal components (PC), principal coordinates (PCo), or lapvectors.

The number of subgroups (from 1 to 15) were examined by MCLUST based on 1-150 PC, PCo, and lapvectors. Our results suggested that the number of subgroups varied between one and nine (Additional file 3). The number of subgroups showed a high variability if less than 20 PC, PCo, or lapvector were used which explained together less than 75% of the variance. However, when the number of PC was higher than 50, the number of subgroups started to vary again (Additional file 4). The explanation for this observation is unclear and requires further research. These findings suggested that determining the number of subgroups using MCLUST applied to PC, PCo, or lapvector is not straight forward and requires careful consideration of the numbers of dimensions used for the analyses.

When the number of subgroups was set to two according to the results of PCA, PCoA, and LAP, we observed for 10-40 PC, 10-50 PCo, and 1-100 lapvectors *>*95% correspondence with the germplasm type information (Additional file 4) and *>*90% correspondence with the assignment by STRUCTURE (data not shown). The above mentioned methods also had with *>*85% a high correspondence of assignment with each other (data not shown). These findings suggested that these methods might be time-saving alternatives to STRUCTURE analyses, if the assignment of genotypes to subgroups is of interest and the numbers of subgroups is known.

### Population structure of the elite sugar beet germplasm

Results of earlier studies revealed that cultivated sugar beet genotypes are genetically distinct from wild beet genotypes [37,9]. Moreover, the results of [6] indicated that the seed and pollen parent heterotic pools of cultivated sugar beet showed two distinct clusters after 40 years of recurrent reciprocal selection. Therefore, in our study, the population structure of one of these two heterotic pools, namely the pollen parent heterotic pool was examined in further detail.

The results of the STRUCTURE analysis revealed the presence of two subgroups in the entire pollen parent germplasm set (Additional file 1). This observation was in accordance with the clustering observed in the PCA, PCoA and LAP analyses as well as with the MCLUST analysis and with the number of examined germplasm types (Figure 2, Additional file 2). Furthermore, 99.6% of the inbreds in the subgroup 1 based on the MCLUST analysis with 10 PCs were sugar types and 98.5% of the inbreds in the subgroup 2 yield types. The observed pattern of population structure might be explained by the fact that due to a negative correlation between root yield and sugar content [7], the selection on both traits in an originally undifferentiated population could lead to differentiated populations. The observation of distinct subgroups was further made possible by the occurrence of only few recombination events between the two germplasm types [8]. Nevertheless, we observed a higher average MRD for all the inbreds than for that between two germplasm types. This observation indicated that higher variation existed within the populations than between the populations.

Our explanation is in accordance with the observation that the IIlinois long term selection experiment for grain protein (high vs. low protein) and oil concentration (high vs. low oil) in maize had lead to phenotypically but also genotypically divergent populations [38]. Due to the fact that germplasm type information was in very good agreement with molecular marker information, sugar type and yield type inbreds were the basis for all further analyses.

### Comparison of different numbers of SNPs for detecting population structure

As the SNP number and selection strategy is expected to affect the estimates of population structure (c.f. [14]), we examined these aspects in our study. The correspondence of assignment by MCLUST based on subsets of 9-252 SNPs vs. the whole SNP set improved with an increasing number of SNPs (Figure 3). Similarly, the *CV *of MRD estimates among all pairs of inbreds decreased with increasing number of SNPs (Figure 4). This is due to the fact that a high number of SNPs provides a high precision for determining population structure as well as for measuring the genetic distance between inbreds. When the SNP numbers selected at random or in a stratified fashion reached about 100, the before mentioned trends of the correspondence as well as the *CV *reached a plateau and not much further improvement could be obtained by further increasing the number of SNPs. As the costs for genotyping will also increase with an increasing number of SNPs, our results indicated that in the examined sugar beet germplasm about 100 SNPs would be required to determine the same population structure as the whole SNPs set did and that this estimation would be done with a similar precision.

We observed a slightly higher correspondence (Figure 3) as well as lower *CV *of MRD (Figure 4) for the stratified than for the random resampling strategy. This observation suggested that by choosing markers that are equally distributed across the genome, it is possible to reduce their number compared to randomly distributed markers while achieving the same level of precision in assigning inbreds to subgroups as well as estimating MRD. An even higher correspondence can be obtained with the same number of markers if they were selected with respect to their PIC values (Figure 3). This observation suggested that with SNPs selected for a high PIC value, the number of SNP markers required to determine the same population structure could be further reduced.

The number of SNPs predicted in our study to be required for MRD estimates is considerably lower than that calculated for maize [12]. This observation might be explained by differences in the number of genotypes studied. [12] examined three times more genotypes than we did, which increases the number of markers required to unambiguously identifying each genotype. Furthermore, [12] examined 25 times more SNPs than we did, which also increases the number of markers required to achieve a similar precision as the whole SNPs set did.

### Genome-wide distribution of genetic diversity

Elite sugar beet germplasm has been intensively selected since the mid of the last century [8]. Consequently, the genomic regions controlling traits of economic importance are expected to be shaped by this selection. Therefore, characterizing the genome-wide distribution of genetic diversity of elite sugar beet germplasm which has been selected for different traits, such as sugar content vs. root yield might help to identify the genes controlling these traits. A similar approach has been successfully applied to identify a panel of known genes as well as some interesting candidate genes and QTLs in Holstein cattle [22].

We observed an average gene diversity of 0.338 for the entire germplasm set. This finding is in good accordance with results of [37] where a gene diversity of 0.31 was observed in USDA sugar beet gene bank materials assessed with RAPD markers. In contrast, the gene diversity observed in our study was lower than the values reported earlier ([26,9,6]), where an average gene diversity of 0.51-0.62 was observed in weed beet and sugar beet populations using SSR markers. This difference might be explained by the examined marker types. SNP and RAPD markers are typically bi-allelic, whereas SSR markers are multi-allelic, which has the potential to increase gene diversity (c.f. [12]).

The average gene diversity of the sugar type inbreds was higher than that of the yield type inbreds (Additional file 5). This observation might be explained by ascertainment bias during SNP development or a higher selection intensity applied during breeding of yield type sugar beets compared to sugar type inbreds. Our explanation was supported by the fact that the effective population size *Ne *of the yield type inbreds was considerably lower than that of the sugar type inbreds (Table 2), which indicated stronger bottleneck effects for the yield types than for the sugar type inbreds. However, it should be noted that the calculation of *Ne *assumes idealized populations [34], and that where these idealizations are violated such as selected populations or selected SNPs, the calculated *Ne *will deviate from the true value. Another reason for our finding of a higher gene diversity of the sugar type inbreds compared to the yield type inbreds might be that it is more difficult to introduce new germplasm from exotic sources into the yield types than into the sugar types.

The unequal distribution of genetic diversity across the genome could be explained by the ascertainment bias during SNP development. However, more likely, this observation is due to the selection history of the different genome regions. Therewith, the genome-wide distribution maps of genetic diversity (Additional file 5 and 6) might be a first step to identify the target genes or regions selected during breeding history. For example, genes related to sugar content and root yield might be present in the most divergent genomic regions between these two germplasm types. Common genes under selection in the breeding program of the both germplasm types (e.g. disease resistant genes) might be present in the genomic regions showing the same level of gene diversity and low MRD (Additional file 5 and 6).

### Genome-wide distribution of LD and consequences for association mapping

The power and resolution of association mapping depend greatly on the genome-wide distribution of LD assessed with a high number of markers [39]. We observed that a total of 18.97%, 31.84%, and 32.01% of the linked loci pairs in the entire germplasm set, yield and sugar type inbreds, respectively, showed *r*^2 ^values higher than the significance threshold (Table 1). The percentages observed in our study were lower than that reported earlier [6]. In contrast, the values of our study were higher than that of earlier studies [26,27,9], where 1.1%-14.3% of the loci paris were observed to be in significant LD. These differences might be explained by the facts that (i) different significance thresholds were used, (ii) a rather high marker density was applied in our study compared to earlier studies, (iii) different marker types were used in these studies, i.e. SNPs in our study vs. SSRs or RAPDs in other studies, and (iv) different plant materials was examined, i.e homozygous elite inbreds of sugar beet in our study and [6] vs. random mating wild beets in other studies.

As *r*^2 ^between SNPs decayed with genetic map distance, we suggest that linkage between SNPs is an important factor influencing the patterns of LD in the studied germplasm. The *r*^2 ^reached the threshold of significant LD within 7.4 cM, 45.1 cM, and 20.6 cM for the entire germplasm set, yield type and sugar type inbreds, respectively. In addition, $\bar{r^{2}}$ at binned genetic map distances reached a plateau at 15-20 cM for the entire gemplasm set and the two germplasm types. The decay distance we observed was longer than that reported by [6], where *r*^2 ^declined to 0.1 at 10 cM, and that of [25] where only marker pairs *<*3 cM showed a high extent of LD. The difference might be due to (i) the rather high density of markers examined in our study compared with earlier studies and (ii) different regression methods used to measure the decay of LD. The observation of slower LD decay for yield type inbreds than for sugar type inbreds, which might be due to the different selection history as outlined above, resulted in smaller effective population sizes *Ne *calculated for the yield type inbreds than the sugar type inbreds (Table 2). The results indicated that different numbers of markers are required for genome-wide association mapping in the different types of germplasm.

The high proportion of SNP loci pairs in significant LD as well as the decay of LD with distance suggested that association mapping is a tool applicable in the context of sugar beet breeding. However, both in the entire germplasm set and the two groups of the germplasm types we observed only for very few (0.74-6.22%) linked SNP paris *r*^2 ^values *>*0.8 (Table 1). Such high *r*^2 ^values are required in order to allow the detection of marker-phenotype associations explaining less than 1% of the phenotypic variance [32]. This in turn indicates that for genome-wide association mapping in sugar beet, the number of markers has to be dramatically increased compared to the number applied in our study.

We observed different LD levels along the linkage groups of sugar beet (Additional file 8). This observation suggests that estimating the number of markers required for genome-wide association mapping from the genome-wide average of LD is dubious. In this case, important QTL might be not detected as locally occuring low levels of LD decrease the power to detect them. Therefore, the genome-wide distribution of LD has to be considered when designing SNP genotyping arrays in the context of genome-wide association mapping. Furthermore, the LD patterns found in the pollen parent heterotic pool might not be the right information source for designing SNP genotyping arrays for other germplasm.

## Conclusions

We identified based on different statistical methods two distinct subgroups in the elite sugar beet germplasm of the pollen parent heterotic pool, which is in accordance with its breeding history. MCLUST based on principal components, principal coordinates, or lapvectors might be an alternative method to STRUCTURE for population structure analysis. Gene diversity and MRD between the examined germplasm types varied considerably across the genome, which might be due to artificial selection. This fact could be used to identify candidate genes for the traits under selection using population genetics tools. Furthermore, similar approaches using sequences of wild and cultivated sugar beet genotypes might be used to identify the domestication genes. Due to the fact that *r*^2 ^*>*0.8 is required to detect marker-phenotype association explaining less than 1% of the phenotypic variance, our observation of a low proportion of SNP loci pairs fulfilling this criterion suggests that the number of markers has to be dramatically increased for genome-wide association mapping.

## Authors' contributions

BS, AKL, and KW conceived and supervised the study. JL and BS analysed and interpreted the data. AKL and KW provided the data. JL and BS wrote the manuscript. All authors read and approved the final manuscript.

## Supplementary Material

Additional file 1 **(a) Log likelihood, (b) Δ*K *values for different number of subgroups (*K*) in the entire germplasm set**. Click here for file

Additional file 2 **Principal coordinate analysis, Laplacian eigenfunctions analysis, and Principal component analysis of the entire elite sugar beet germplasm set**. (a) Principal coordinate analysis based on modified Roger's distance (MRD) estimates, (b) Laplacian eigenfunctions analysis, and (c) Principal component analysis of the entire elite sugar beet germplasm set. PC 1 and PC 2 refer to the first and second principal components/coordinates, respectively. LAP 1 and LAP 2 refer to the first and second lapvectors, respectively. The numbers in parentheses refer to the proportion of variance explained by the corresponding axes. Symbols identify the germplasm types and colors the STRUCTURE subgroups. SG 1 and SG 2 are the two subgroups identified by STRUCTURE based on the maximum membership probability threshold. Click here for file

Additional file 3 **Number of subgroups identified by MCLUST**. Number of subgroups identified by MCLUST based on different numbers of (a) principal components, (b) principal coordinates, and (c) lapvectors, and the cumulative proportion of explained variance of (d) principal components, (e) principal coordinates, and (f) lapvectors. Click here for file

Additional file 4 **Correspondence between the known germplasm types of the sugar beet inbreds and the assignment by MCLUST**. Correspondence between the known germplasm types of the sugar beet inbreds and the assignment by MCLUST based on different numbers of (a) principal components, (b) principal coordinates, and (c) lapvectors when the number of subgroups was set to two. Click here for file

Additional file 5 **Genome-wide distribution of gene diversity of yield and sugar type inbreds**. Green and red lines indicate gene diversity of yield and sugar type inbreds, respectively. Dashed lines indicate the average gene diversity of the corresponding germplasm type. Vertical lines at each point indicate standard error multiplied by 100 which were calculated by bootstrapping across genotypes. Vertical lines at the x axis indicate genetic map positions of the SNP loci on the nine linkage groups. Click here for file

Additional file 6 **Modified Roger's distance (MRD) between yield and sugar type inbreds across the genome**. Dashed lines indicate average MRD across the genome and dotted lines average MRD for each linkage group. Vertical lines at each point represent the standard error multiplied by 10 which were calculated by bootstrapping across genotypes. Vertical lines at the x axis indicate genetic map positions of the SNP loci on the nine linkage groups. Click here for file

Additional file 7 **Plot of linkage disequilibrium measured as squared correlation of allele frequencies (*r*^2^) against genetic map distance (cM) between linked loci pairs**. (a) yield type and (b) sugar type inbreds. The red line is the nonlinear regression trend line of *r*^2 ^vs. genetic map distance. The dashed line indicates the 95% quantile of *r*^2 ^between unlinked loci pairs. Click here for file

Additional file 8 **Average linkage disequilibrium measured as squared correlation of allele frequencies (*r*^2^) for all linked loci pairs within 5 cM segments across the genome**. Green and red lines indicate average *r*^2 ^for yield and sugar type inbreds, respectively. The vertical line at each point represents the standard error. Click here for file

## References

[B1] Grimmer MK Trybush S Hanley S Francis SA Karp A Asher MJC An anchored linkage map for sugar beet based on AFLP, SNP and RAPD markers and QTL mapping of a new source of resistance to beet necrotic yellow vein virus Theoretical and Applied Genetics 2007 114 1151 1160 10.1007/s00122-007-0507-3 17333102

[B2] Draycott AP Sugar beet 2006 Blackwell Publishing Ltd

[B3] Arumuganathan K Earle ED Nuclear DNA content of some important plant species Plant Molecular Biology Reporter 1991 9 415 415

[B4] Lange C Holtgraewe D Schulz B Weisshaar B Himmelbauer H Construction and characterization of a sugar beet (*Beta vulgaris*) fosmid library Genome 2008 51 948 951 10.1139/G08-071 18956027

[B5] Biancardi EC Larry GS George NB Marco D Genetics and breeding of sugar beet 2005 Edenbridge Limited

[B6] Li J Schulz B Stich B Population structure and genetic diversity in elite sugar beet germplasm investigated with SSR markers Euphytica 2010 175 35 42 10.1007/s10681-010-0161-8

[B7] Hendriksen AJT van der Have FDJ Growers RS Kappelle-Biezelinge M The use of some correaltions in beet breeding Euphytica 1953 2 1 5

[B8] Bosemark NO Genetics and breeding 2006 chap 4 Blackwell Publishing Ltd 50 83

[B9] Andersen NS Siegismund HR Meyer V Jorgensen RB Low level of gene flow from cultivated beets (*Beta vulgaris *L. ssp *vulgaris*) into Danish populations of sea beet (*Beta vulgaris *L. ssp. *maritima *(L.) Arcangeli) Molecular Ecology 2005 14 1391 1405 10.1111/j.1365-294X.2005.02490.x 15813779

[B10] Rafalski A Applications of single nucleotide polymorphisms in crop genetics Curr Opin Plant Biol 2002 5 94 100 10.1016/S1369-5266(02)00240-6 11856602

[B11] Syvänen AC Accessing genetic variation: genotyping single nucleotide polymorphisms Nature Review Genetics 2002 2 930 942 10.1038/35103535 11733746

[B12] Van Inghelandt D Melchinger AE Lebreton C Stich B Population structure and genetic diversity in a commercial maize breeding program assessed with SSR and SNP markers Theoretical and Applied Genetics 2010 120 1289 1299 10.1007/s00122-009-1256-2 20063144 PMC2854351

[B13] McCouch SR Zhao K Wight M Tung CW Ebana K Thomson M Reynolds A Wang D DeClerck G Ali ML McClung A Eizenga G Bustamante C Development of genome-wide SNP assays for rice Breeding Science 2010 60 524 535 10.1270/jsbbs.60.524

[B14] Moragues M Comadran J Waugh R Milne I Flavell AJ Russell JR Effects of ascertainment bias and marker number on estimations of barley diversity from high-throughput SNP genotype data Theoretical and Applied Genetics 2010 120 1525 1534 10.1007/s00122-010-1273-1 20157694

[B15] Van K Hwang EY Kim MY Park HJ Lee SH Cregan PB Discovery of SNPs in soybean genotypes frequently used as the parents of mapping populations in the United States and Korea Journal of Heredity 2005 96 529 535 10.1093/jhered/esi069 15994422

[B16] Schneider K Kulosa D Soerensen TR Moehring S Heine M Durstewitz G Polley A Weber E Lein J Hohmann U Tahiro E Weisshaar B Schulz B Koch G Jung C Ganal M Analysis of DNA polymorphisms in sugar beet (*Beta vulgaris *L.) and development of an SNP-based map of expressed genes Theoretical and Applied Genetics 2007 115 601 615 10.1007/s00122-007-0591-4 17622508

[B17] Pritchard JK Stephens M Donnelly P Inference of population structure using multilocus genotype data Genetics 2000 155 945 959 10835412 10.1093/genetics/155.2.945 PMC1461096

[B18] Pearson K On lines and planes of closest fit to system of points in space Philosophical Magazine 1901 2 559 572

[B19] Gower JC Some distance properties of latent root and vector methods used in multivariate analysis Biometrika 1966 53 325 338

[B20] Zhang J Niyogi P McPeek MS Laplacian eigenfunctions learn population structure PLoS ONE 2009 4 e7928 10.1371/journal.pone.0007928 19956572 PMC2779848

[B21] Fraley C Raftery AE Model-based methods of classification: Using the MCLUSTt software in chemometrics Journal of Statistical Software 2007 18 1 13

[B22] Qanbari S Pimentel ECG Tetens J Thaller G Lichtner P Sharifi AR Simianer H A genome-wide scan for signatures of recent selection in Holstein cattle Anim Genet 2010 41 377 89 20096028 10.1111/j.1365-2052.2009.02016.x

[B23] Stich B Melchinger AE Heckenberger M Möhring J Schechert A Piepho HP Association mapping in multiple segregating populations of sugar beet (*Beta vulgaris *L.) Theoretical and Applied Genetics 2008 117 1167 1179 10.1007/s00122-008-0854-8 18719879

[B24] Stich B Piepho HP Schulz B Melchinger AE Multi-trait association mapping in sugar beet (*Beta vulgaris *L.) Theoretical and Applied Genetics 2008 117 947 954 10.1007/s00122-008-0834-z 18651127

[B25] Kraft T Hansen M Nilsson NO Linkage disequilibrium and fingerprinting in sugar beet Theoretical and Applied Genetics 2000 101 323 326 10.1007/s001220051486

[B26] Arnaud JF Fénart S Godé C Deledicque S Touzet P Cuguen J Fine-scale geographical structure of genetic diversity in inland wild beet populations Molecular Ecology 2009 18 3201 15 10.1111/j.1365-294X.2009.04279.x 19627487

[B27] Viard F Arnaud JF Delescluse M Cuguen J Tracing back seed and pollen flow within the crop-wild *Beta vulgaris *complex: genetic distinctiveness vs. hot spots of hybridization over a regional scale Molecular Ecology 2004 13 1357 1364 10.1111/j.1365-294X.2004.02150.x 15140082

[B28] Evanno G Regnaut S Goudet J Detecting the number of clusters of individuals using the software STRUCTURE: A simulation study Molecular Ecology 2005 14 2611 2620 10.1111/j.1365-294X.2005.02553.x 15969739

[B29] Rosenberg NA Distruct: a program for the graphical display of population structure Molecular Ecology Notes 2004 4 137 138

[B30] Patterson N Price AL Reich D Population structure and eigenanalysis PLoS Genetics 2006 2 e190 10.1371/journal.pgen.0020190 17194218 PMC1713260

[B31] Wright S Evolution and genetics of populations 1978 IV The University of Chicago Press, Chicago

[B32] Ersoz ES Yu J Buckler ES Application of linkage disequilibrium and association mapping in maize 2009 Charter 13 Springer-Verlag Berlin Heidelberg chap 173 195

[B33] Heuertz M Emanuele DP Källman T Larsson H Jurman I Morgante M Lascoux M Gyllenstrand N Multilocus patterns of nucleotide diversity, linkage disequilibrium and demographic history of Norway spruce (*Picea abies *(L.) Karst) Genetics 2006 174 2095 2105 10.1534/genetics.106.065102 17057229 PMC1698656

[B34] Hill WG Weir BS Variances and covariances of squared linkage disequilibria in finite populations Theoretical and Applied Genetics 1988 33 54 78 10.1016/0040-5809(88)90004-4 3376052

[B35] R Development Core Team R: A Language and Environment for Statistical Computing 2011 R Foundation for Statistical Computing, Vienna, Austria

[B36] Price AL Patterson NJ Plenge RM Weinblatt ME Shadick NA Reich D Principal components analysis corrects for stratification in genome-wide association studies Nature Genetics 2006 38 904 909 10.1038/ng1847 16862161

[B37] McGrath JM Derrico CA Yu Y Genetic diversity in selected, historical US sugarbeet germplasm and *Beta vulgaris ssp. maritima* Theoretical and Applied Genetics 1999 98 968 976 10.1007/s001220051157

[B38] Moose SP Dudley JW Rocheford TR Maize selection passes the century mark: a unique resource for 21st century genomics Trends in Plant Science 2004 9 358 364 10.1016/j.tplants.2004.05.005 15231281

[B39] Stich B Melchinger AE Frisch M Maurer HP Heckenberger M Reif JC Linkage disequilibrium in European elite maize germplasm investigated with SSRs Theoretical and Applied Genetics 2005 111 723 730 10.1007/s00122-005-2057-x 15997389

