# Supplementary figures and images for: Genome-wide distribution of genetic diversity and linkage disequilibrium in elite sugar beet germplasm
Source: BMC Genomics. 2011 Oct 4;12:484. doi: 10.1186/1471-2164-12-484 (PMC3213064; doi:10.1186/1471-2164-12-484)

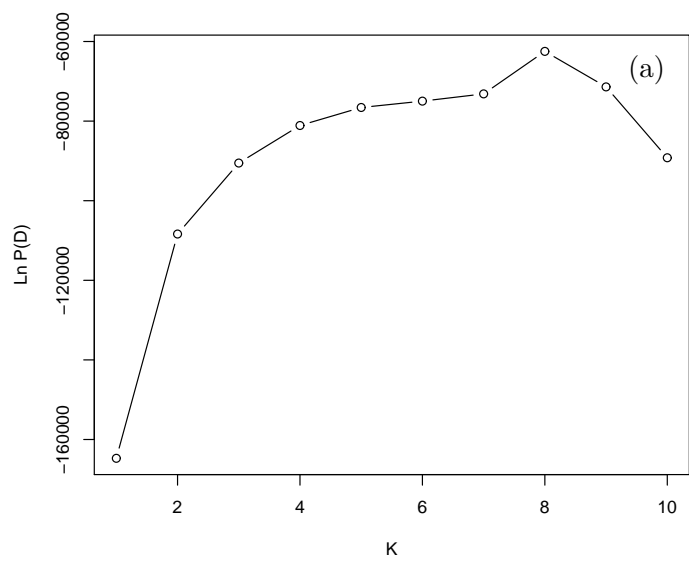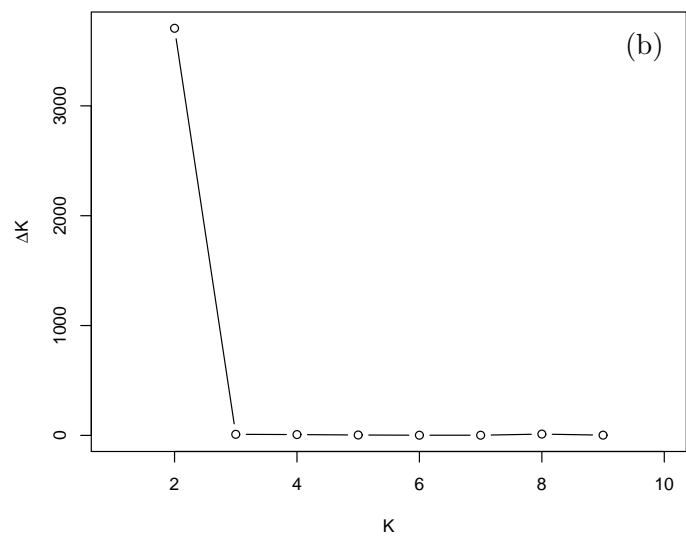

Supplement: Additional file 1 — (a) Log likelihood, (b) ΔK values for different number of subgroups (K) in the entire germplasm set. [file 1471-2164-12-484-S1.PDF]

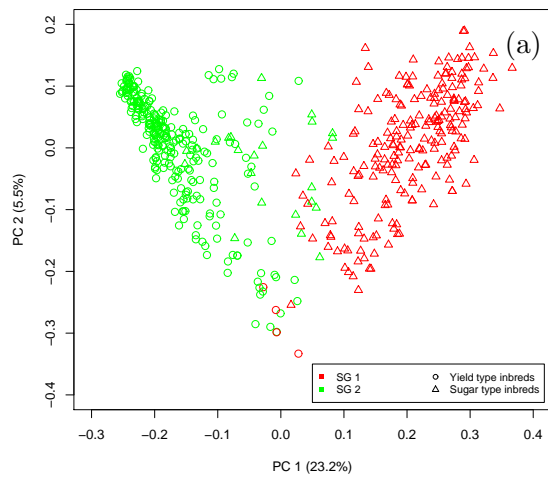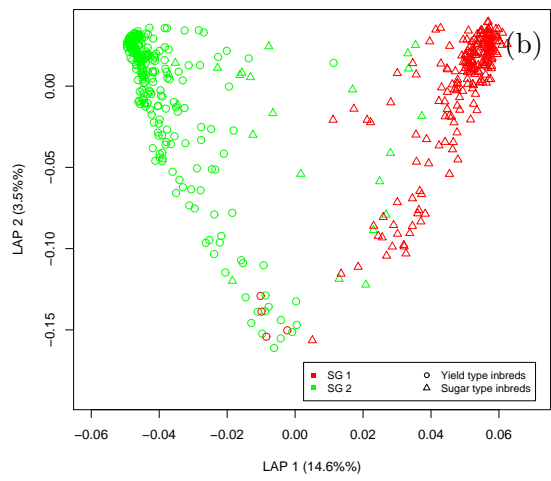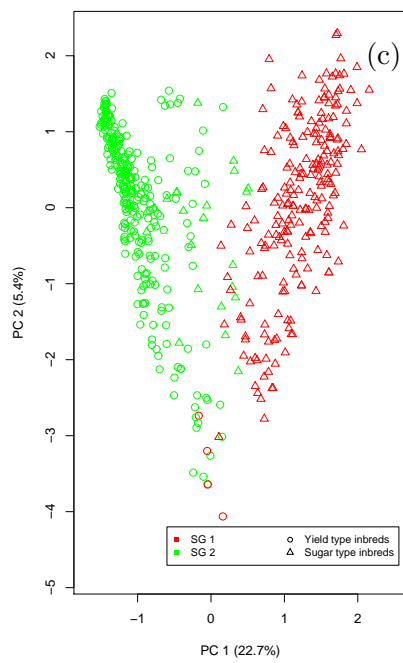

Supplement: Additional file 2 — Principal coordinate analysis, Laplacian eigenfunctions analysis, and Principal component analysis of the entire elite sugar beet germplasm set. (a) Principal coordinate analysis based on modified Roger's distance (MRD) estimates, (b) Laplacian eigenfunctions analysis, and (c) Principal component analysis of the entire elite sugar beet germplasm set. PC 1 and PC 2 refer to the first and second principal components/coordinates, respectively. LAP 1 and LAP 2 refer to the first and second lapvectors, respectively. The numbers in parentheses refer to the proportion of variance explained by the corresponding axes. Symbols identify the germplasm types and colors the STRUCTURE subgroups. SG 1 and SG 2 are the two subgroups identified by STRUCTURE based on the maximum membership probability threshold. [file 1471-2164-12-484-S2.PDF]

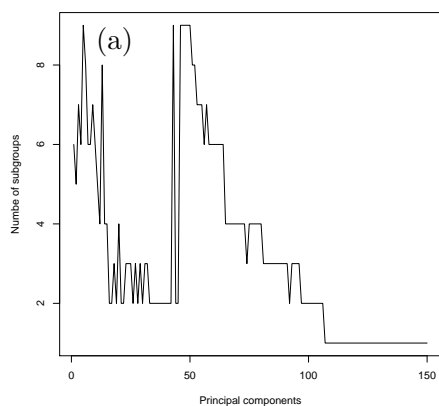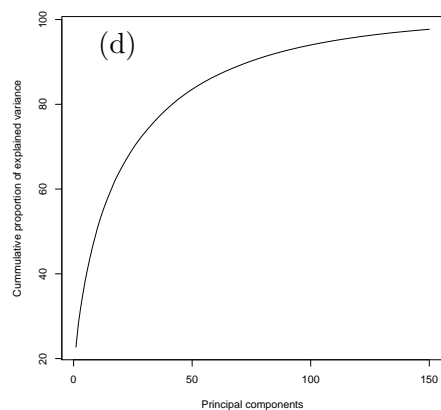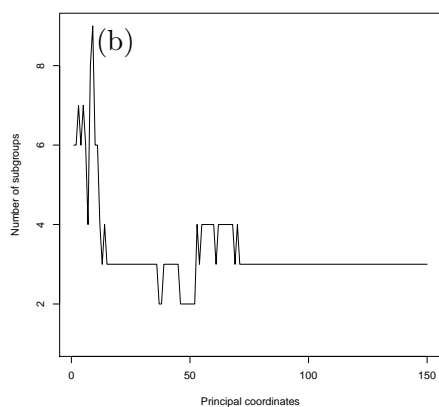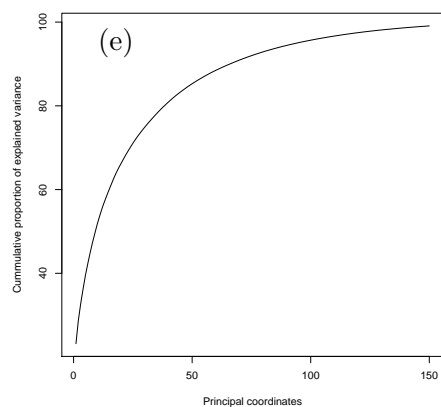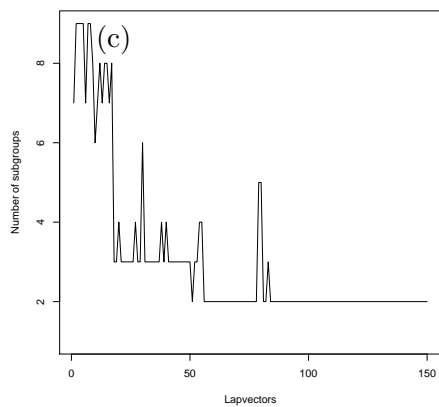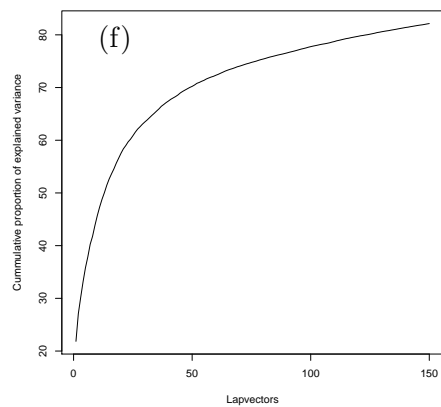

Supplement: Additional file 3 — Number of subgroups identified by MCLUST. Number of subgroups identified by MCLUST based on different numbers of (a) principal components, (b) principal coordinates, and (c) lapvectors, and the cumulative proportion of explained variance of (d) principal components, (e) principal coordinates, and (f) lapvectors. [file 1471-2164-12-484-S3.PDF]

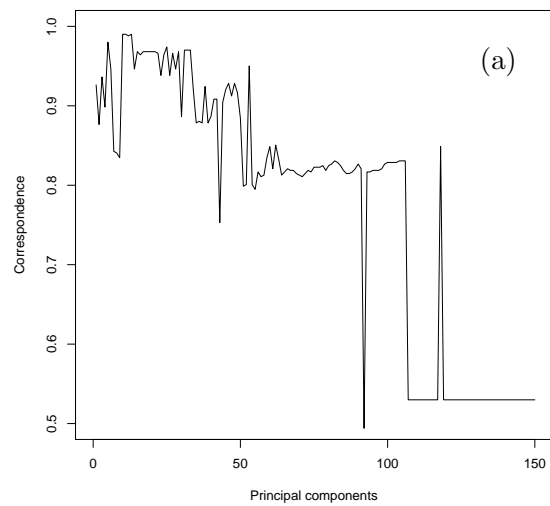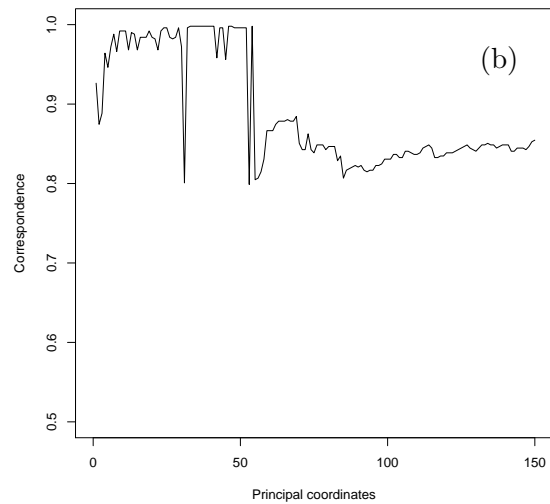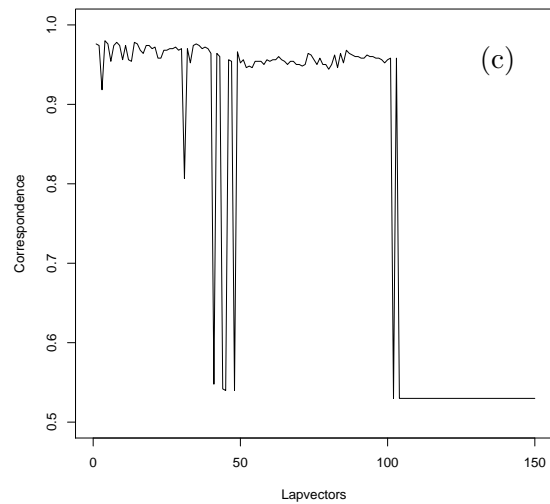

Supplement: Additional file 4 — Correspondence between the known germplasm types of the sugar beet inbreds and the assignment by MCLUST. Correspondence between the known germplasm types of the sugar beet inbreds and the assignment by MCLUST based on different numbers of (a) principal components, (b) principal coordinates, and (c) lapvectors when the number of subgroups was set to two. [file 1471-2164-12-484-S4.PDF]

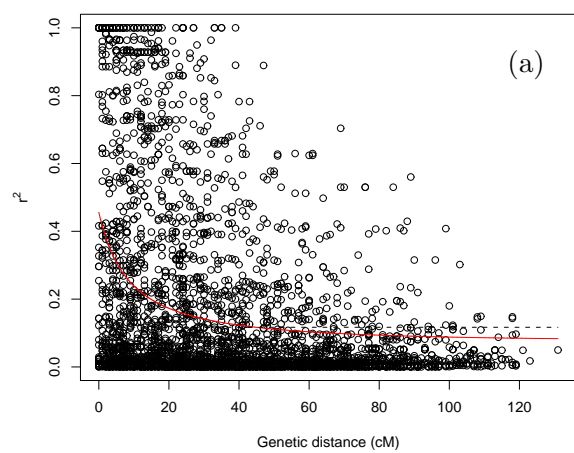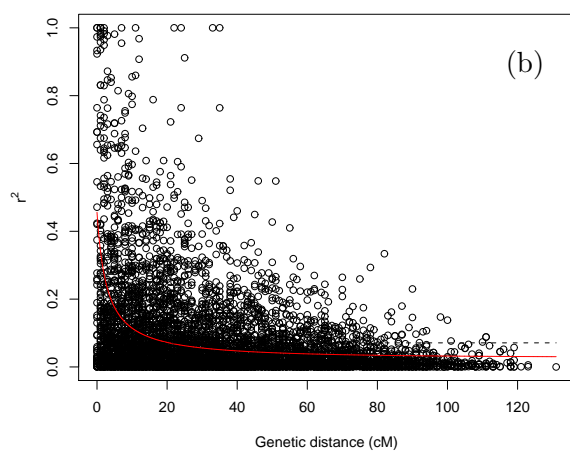

Supplement: Additional file 7 — Plot of linkage disequilibrium measured as squared correlation of allele frequencies (r2) against genetic map distance (cM) between linked loci pairs. (a) yield type and (b) sugar type inbreds. The red line is the nonlinear regression trend line of r2 vs. genetic map distance. The dashed line indicates the 95% quantile of r2 between unlinked loci pairs. [file 1471-2164-12-484-S7.PDF]
